# Response to “Scrutinizing the causal link between excited delirium syndrome and restraint – a commentary on: ‘The role of restraint in fatal excited delirium: a research synthesis and pooled analysis’ by E.M.F. Strömmer, W. Leith, M.P. Zeegers and M.D. Freeman”

**DOI:** 10.1007/s12024-023-00616-3

**Published:** 2023-04-26

**Authors:** Michael D. Freeman, Ellen M. F. Strömmer, Wendy M. Leith, Maurice P. Zeegers

**Affiliations:** 1 Faculty of Health, Medicine, and Life Sciences, Maastricht University, Maastricht, Netherlands; 2 PO Box 96309, Portland, OR USA

**Keywords:** Excited Delirium, Agitated Delirium, Circular Reasoning, Bias, Logical Fallacy

## Abstract

de Boer et al. criticize the conclusions in our 2020 paper on the validity of Excited Delirium Syndrome (ExDS) as “*egregiously misleading*.” Our conclusion was that there “*is no existing evidence that indicates that ExDS is inherently lethal in the absence of aggressive restraint*.” The basis for de Boer and colleague’s criticism of our paper is that the ExDS literature does not provide an unbiased view of the lethality of the condition, and therefore the true epidemiologic features of ExDS cannot be determined from what has been published. The criticism is unrelated to the goals or methods of the study, however. Our stated purpose was to investigate “*how the term ExDS has evolved in the literature and been endowed with a uniquely lethal quality*,” and whether there is “*evidence for ExDS as a unique cause of a death that would have occurred regardless of restraint, or a label used when a restrained and agitated person dies, and which erroneously directs attention away from the role of restraint in explaining the death*.” We cannot fathom how de Boer et al. missed this clearly stated description of the study rationale, or why they would endorse a series of fallacious and meaningless claims that gave the appearance that they failed to grasp the basic design of the study. We do acknowledge and thank these authors for pointing out 3 minor citation errors and an equally minor table formatting error (neither of which altered the reported results and conclusions in the slightest), however.

We read the letter from de Boer et al. [1] with interest. While we acknowledge the great lengths these authors have gone to in an attempt to refute the findings in our 2020 paper titled “The role of restraint in fatal excited delirium: a research synthesis and pooled analysis,” [2] it is readily apparent that they failed to grasp the clearly stated purpose and design of the study, and instead engaged in a series of fallacious arguments and misrepresentations of the methods, findings, and conclusions of the study.

As we described in our paper, we set out to review the world literature regarding cases of fatal and survived excited delirium syndrome and agitated delirium (abbreviated as ExDS and AgDS, respectively), described either individually or as part of a group. The purpose of the study was to evaluate the evidence for ExDS as a unique pathophysiologic cause of death, versus whether the term may instead serve as a proxy for restraint-related death. We made this goal abundantly clear when we stated, in the introduction to our paper, the following:

*Investigation of the potential for ****circular reasoning ****in how the term ExDS has evolved in the literature and been endowed with a uniquely lethal quality is the primary aim of this paper, as we attempt to evaluate whether there is evidence for ExDS as a unique cause of a death that would have occurred regardless of restraint, or a label used when a restrained and agitated person dies, and which erroneously directs attention away from the role of restraint in explaining the death.* (emphasis added)

de Boer et al. in turn accuse us of circular reasoning for failing to note that the two sources of cases of ExDS and AgDS in the literature (*i.e.,* either from forensic pathology sources where all of the subjects are deceased, or from clinical sources, where either fatal or survived cases may be recorded) resulted in a hopelessly biased assortment of individual cases upon which we performed our pooled case analysis. Inexplicably, de Boer et al. fault us for doing *precisely what we set out to do*, which was to examine the ExDS/ AgDS literature for evidence that ExDS is a stand-alone cause of death in the absence of restraint. We did not, as de Boer et al. incorrectly infer, attempt to perform an epidemiologic study of the general population diagnosed with ExDS or AgDS, a technically impossible feat, as there are, at present, no means of surveillance of the frequency, settings, circumstances, or outcomes in which the diagnoses may be applied. As noted in our paper, ExDS does not exist in the International Classification of Diseases coding, and therefore hospital and Centers for Disease Control Multiple Cause of Death databases do not contain any mention of the diagnosis. Thus, case studies and case series are the sole source of descriptions of the circumstances and outcomes in which either ExDS or AgDS has been diagnosed. We clearly stated in the “limitations” section of our discussion (more on this below) that the nature of the literature on ExDS results in a biased selection of cases; this is just basic epidemiologic common sense. Our goal was to investigate a critical question regarding the “chicken or egg” nature of an ExDS diagnosis in the context of death occurring during restraint, which is “Does ExDS cause death, or does death cause ExDS?”

Our literature review results were divided into individually described cases, and cases described as part of a group. Only the individually described cases, totaling 168, were included for regression analysis, as the group studies were lacking in sufficient detail to be able to draw any meaningful conclusions regarding the interaction of restraint, diagnosis (ExDS or AgDS), and death as an outcome. Once we identified the individual cases that fit the inclusion criteria, we described the case characteristics and analyzed the predictive features of fatal versus non-fatal cases, as well as a diagnosis ExDS vs. AgDS. We found that nearly all of the ExDS cases that were fatal also had some form of restraint described, and, most importantly, that risk of death increased monotonically with the aggressivity of the described restraint methods. We concluded that **in the absence of restraint, there is no evidence that ExDS is a fatal condition**. Given that, on the one hand, there are no known pathological mechanisms linking a diagnosis of ExDS to sudden death, and on the other hand there is strong evidence that weighted prone restraint is associated with restricted respiration and thus a risk of asphyxia, we concluded that, “When death has occurred in an aggressively restrained individual who fits the profile of ExDS or AgDS, restraint-related asphyxia must be considered a likely cause of the death.”

de Boer et al. claimed that this conclusion was not only unsupported by our methods, but that it was “egregiously misleading.” This is an apt description for the majority of the arguments and all of the conclusions in the commentary by de Boer et al. Our finding that the majority of ExDS cases were fatal and AgDS cases were not is not a failing of the study design, but rather *an artifact of what has been published in the literature*. The irrefutable conclusion that fatal ExDS is described in the world literature as nearly always occurring in the context of restraint, and that, in absence of restraint, there is no evidence that ExDS is fatal, or that when the condition is survived in a clinical setting it is more likely to be deemed AgDS, apparently escaped the notice of de Boer et al. in their haste to criticize our methods, data, and conclusions.

de Boer et al. focused on 3 points in their letter:

1. The conclusions in our paper used circular reasoning to interpret the findings.
2. The references used in the paper were “unsuited” to its aims.
3. The errors in citations were sufficient to discredit the entire paper.

In the following section of this response is a more detailed refutation of each point:

1. *The conclusions in our paper used circular reasoning to interpret the findings.*

At the forefront of de Boer et al.’s critique of our paper is that, due to the biased nature of the literature on ExDS cases, any conclusions are inherently subject to circular reasoning. de Boer et al. reviewed approximately 80 of 103 citations from our paper and categorized the data source for each paper as either clinical (*i.e.,* EMT or emergency medicine) or non-clinical (*i.e.*, forensic pathology). de Boer and colleagues concluded:

Based on our findings, it should not be surprising that a diagnosis of ExDS was more often fatal and more often associated with unknown restraint or sedation…

*Several papers only focused on fatal cases…This case selection bias [was] inherent in the literature … Due to circular reasoning, such papers cannot be used to ****study the lethality ****of ExDS/AgDS.* (emphasis added)

Again, we note that de Boer et al. demonstrate a failure to comprehend the basic design of our study. Our research goal was never to provide epidemiologic evidence of the lethality of ExDS/ AgDS (*i.e.*, the risk of death secondary to the conditions, which would require an epidemiologic study which, as noted above, is not possible), but rather to examine the evidence for ExDS as a cause of death in the absence of the competing cause of restraint-related asphyxia. Our analysis was quite obviously not performed on a random sample of cases of ExDS/ AgDS, but rather it was a comprehensive review of all papers published in the scientific literature that describe cases of ExDS or AgDS. The literature is what it is, and our reporting and analysis of published cases was not influenced by a biased selection of the literature on our part (as we plainly stated and adhered to the criteria for our review), but rather a fair and unbiased reporting of what actually exists in the literature.

Rather bizarrely, and despite their accusations, de Boer et al. state that they were aware that we accounted for the biased reporting of fatal ExDS cases in the literature, as they admit that:

*It appears that Strömmer et al. were aware of the…shortcoming, because in the introduction they discuss the different use of the terms [ExDS and AgDS]. ****In the discussion they also mention the limitation that fatal cases are more often published and are more detailed in terms of type of restraint. ****However, they fail to recognize how the available information differs between ExDS and AgDS, and how their results are ****confounded ****by it.* (emphasis added)

It is possible that de Boer et al. failed to carefully read our paper when they made this fanciful and false allegation, as in the discussion we clearly stated:

*Thus, the evidence suggests that ExDS is not a unique cause of death in the absence of restraint, and that the supposition to the contrary is an artifact of circular reasoning and ****confounding ****rather than an evidence-based inference. While it is possible that ExDS is a fatal condition in the absence of restraint, there is no observational evidence to support this hypothesis in the biomedical literature at the present time.* (emphasis added)

How de Boer et al. managed to note, cite, and then utterly disregard our caveats regarding both selection and information bias in the literature is unclear; we described potential issues with the literature appropriately and thoroughly, and the allegation that we did not “recognize” issues with the literature is a blatant falsehood. Below is what we wrote in the “Discussion” section about limitations and bias in the literature that we reviewed:

*There are a number of limitations to consider when examining and comparing the ExDS and AgDS literature, and the data that can be abstracted from it. The first is that it is ****not possible to capture a representative sample of cases of ExDS or AgDS in national hospital databases ****because neither diagnosis is specified in the ICD-9 or ICD-10, which such databases rely on, and the diagnoses are therefore unsearchable in any of the databases.*

*Second*, ***fatal cases are likely overrepresented in the literature because they are more likely to be written about than survived cases. Fatal cases are also more likely to have more detailed information about the circumstances leading to the death because of the nature of death investigation, which typically involves toxicology screens, police reports, and witness accounts.***

*Third*, ***the cases reported in the literature are not a random sample of either AgDS or ExDS, although deaths are likely overrepresented***.

*Fourth, there is often ****a lack of detail about some of the predictive factors***, *including which drugs (if any) were used, the quantity and combination of drugs used, what level of tolerance an individual may have had to the involved drugs, and what is considered a drug overdose versus contributory to the death.*

*Fifth*, ***details of restraint are also often missing***, *including the type, duration, and force of restraint used, where compression may have occurred (i.e., face, neck, chest, appendages), and whether cardiorespiratory collapse occurred during restraint or at a later time.* (emphasis added)

de Boer et al. also make the following false attribution to our paper:

*When Strömmer et al. use this data to infer that ‘the most probable mechanism driving the ****association ****between ExDS and death is the high frequency of aggressive restraint types observed in the ExDS cases’… they erroneously infer ****causation***. (emphasis added)

Here, the authors engage in a classic “straw man argument,” by attributing to our words a conclusion that we inarguably did not draw. This is not science; it is logical fallacy and misrepresentation of a published work. This is clear from the fact that de Boer et al. acknowledge we only use the term “association,” which in and of itself does not imply causation, a word that was not used once in the paper. Rather, we provided a reasoned and evidence-based explanation for the observed association in our findings, in the context of the weaknesses of the literature. As we stated in our discussion:

*The limitations of the literature are bidirectional: while they ****limit the ability to conclusively state ****that a majority of ExDS deaths are due to restraint, they also ****prohibit the inference ****that the ExDS-related death must be due to anything other than the described restraint that directly preceded cardiorespiratory arrest.* (emphasis added)

While it is unclear how de Boer et al. yet again missed a prominent part of our discussion in making yet another false accusation, their suggestion of a causal association between ExDS fatalities and restraint is well supported by generally accepted methods for assessing such relationships. Recent studies regarding the pathophysiology of restraint-related asphyxia, along with the results of our study, have helped satisfy multiple Bradford-Hill causal criteria, including strength, consistency, specificity, temporality, dose-response relationship, and plausibility of the association [3–6]. Although we did not discuss the Bradford-Hill criteria in the article, de Boer et al.’s attempt to disparage our paper on the grounds of a “correlation versus causation” argument highlights how conservatively we described the association in the original study. We could have easily devoted an expanded section of the paper to a discussion of the evidence for a causal association between ExDS and sudden death in the absence of restraint (*i.e.*, little to none), versus the abundant evidence for a causal relationship between prone restraint and sudden death, not to mention the common-sense link between increased restriction of respiration and increased risk of asphyxia.

2. *The references used in the paper were “unsuited” to its aims.*

de Boer and colleagues re-reviewed some of the literature from which our data were abstracted, and presented a table of their findings, concluding,

*Many of the cited references are totally unsuited to analyze the role of restraint in fatal excited delirium… only a very small portion of the papers provide a comprehensive and detailed discussion needed for the comparative study that Strömmer et al. attempted.*

Once again, de Boer et al. engage in a “straw man” argument by attributing study design and methods to our paper that were manufactured in their imaginations, rather than what was plainly stated in our paper, and then critiquing their own misquotation. We abstracted individual cases of ExDS and AgDS from all of the published English literature in order to create a database of what is in the literature, regardless of presence or absence of details regarding restraint (our inclusion and exclusion criteria for case inclusion were prominently featured in the “Methods” section of the paper). Any paper that adequately described a case of ExDS or AgDS was suited for inclusion, regardless of the overall aim of the source paper (*e.g.,* a discussion of pharmacology of drugs, etc.). de Boer et al. devoted substantial time to categorizing 80 of our cited studies into clinical and non-clinical sources, a meaningless exercise, in part because we did describe the source of information for the circumstances of either diagnosis or outcome in our univariate analysis (*i.e.*, law enforcement, paramedic, or other), but also because it was not our stated intent to dichotomize the data by setting of diagnosis, but rather by outcome, and ExDS vs. AgDS. de Boer et al. are welcome to perform a different study than what we described and publish their results, but the criticism that our study conclusions are “egregiously misleading” because they think we should have conducted a different study is not intellectually honest.

3. *The errors in citations were sufficient to discredit the entire paper*.

In their detailed review and table of 80 of the references used in our study, de Boer et al. asserted that “*Many references were interpreted incorrectly*.” They listed each of the individual cases abstracted for the pooled analysis that they deemed to have been incorrectly interpreted, including 9 citations from the individual analysis (out of 62 total), 3 of which were exceedingly minor citation errors (which we acknowledge), and 6 of which the authors claimed were misclassified (all of which we dispute), as detailed below.

*de Boer et al.*:

*Atherton et al. is cited as if it contributes two cases, but the paper discusses four drug-related deaths of which only one displayed ‘classic CNS-stimulant induced erratic behavior before being found dead’ (no restraint). No diagnosis of ExDS or AgDS was made.*

Response:

Excited delirium is listed in the keywords of the Atherton article, and the term was relevant to the described cases [7]. The single case that de Boer et al. argue should not have been included described unknown restraint (which is how it was coded for analysis) in an individual with a toxic level of methamphetamine, cocaine, and synthetic cathinones (bath salts), which the authors describe as causing "agitation, aggression, violence, and hallucinations." We disagree that this case should have been excluded.

*de Boer et al.*:

*Byard is cited as if it contributes one case, whilst this paper is an editorial on ExDS without case information.*

Response:

The Byard paper was mis-cited, as Byard is a prolific author on the topic of ExDS who is cited more than once in our paper, and thus a different paper published by the same author was the intended citation. The correct citation is *Confluent muscle pallor: a macroscopic marker of cocaine-induced rhabdomyolysis* [8]. We thank de Boer et al. for their detailed review highlighting this minor citation error. The miscitation did not change the case count, categorization used for the analyses, the results of the analyses, or conclusions of the paper.

*de Boer et al.*:

*Kodikara et al. is cited as if it contributes two cases, but it presents a death due to pulmonary embolism and a death due to cardiac tamponade, initially thought to be ExDS. This paper should therefore have been excluded.*

Response:

We disagree with the authors’ claim. Both cases in the Kodikara study had presumptive ExDS, and therefore fit the inclusion criteria for the pooled analysis [9]. The fact that both decedents were found to have other conditions at autopsy does not mean that they should have been excluded from the study.

*de Boer et al.*:

*McDaniel et al. is cited as if it contributes two cases. The paper discusses the clinical effects of GHB withdrawal and states that three of the five patients had severe symptoms, ‘including delirium’. The terms ExDS or AgDS are not used.*

Response:

The two cases used from the McDaniel et al. article were instances in which the case was described as “agitated” and “delusional” [10]. Both cases were included in the AgDS category for this reason, and we disagree that they should have been excluded. If, however, we had excluded them from the analysis, it would have only strengthened the statistical association between fatal ExDS and restraint, as both AgDS cases survived and were restrained. We disagree that the 3rd GHB withdrawal case should have been included, as they were not described as agitated.

*de Boer et al.*:

*O’Halloran and Frank is cited as if it contributes 20 cases, but according to the paper ‘excited (agitated) delirium, [..] could be interpreted as present in all but 3 of the 21 cases’. In five individuals excited or agitated delirium was included in the cause of death.*

Response:

We disagree with this interpretation. “Excited delirium” was listed in the keywords of the O’Halloran and Frank article [11]. As pointed out by de Boer et al., the source article stated that up to 18 of the cases out of the 20 in our study “could be interpreted as” ExDS/ AgDS. The authors do not specify which cases might or might not be interpreted as ExDS. We found that 20 out of 21 cases included descriptions consistent with ExDS/ AgDS, and we disagree with de Boer et al. that two of the cases should have been excluded, as there is nothing mentioned in the paper that would allow for such discrimination.

*de Boer et al.*:

*Penders et al. is cited as if it contributes three cases, but the paper only very briefly mentions 16 cases who displayed ‘some degree of agitation’ following use of methylenedioxypyrovalerone (MDPV). No detailed case information is provided.*

Response:

Like the Byard et al. paper above, the Penders et al. article was miscited, as it was one of two ExDS articles published by the first author in the same year. The correct citation is *Excited delirium following the use of synthetic cathinones (bath salts)* [12]. We thank de Boer et al. for their detailed review highlighting this minor citation error; however, the miscitation did not change the case count or categorization used for the analyses, much less alter the results of the analyses, or conclusions in the study.

*de Boer et al.*:

*Samuel et al. [20] is cited as if it contributes one case, but the paper discusses four cases, of which at least two should have been included (one fatal and one non-fatal case of ExDS).*

Response:

The second case in the Samuel et al. article was initially excluded from analysis because it was described as a man “in a state of excited delirium following intrathecal administration of an analgesic for chronic pain” [13]. As stated in the “Methods” section of our study, cases of “emergence delirium,” occurring typically as a patient in a clinical setting emerges from anesthetic, were excluded from our analysis. The circumstances described for this case were initially deemed to overlap too closely with emergence delirium to be included, as intrathecal analgesia is a form of anesthesia. Upon revisiting the Samuel et al. article and locating the source article for the case by Levin et al. [14], we found that, to complicate matters further, the case was originally described as AgDS, and that the patient’s condition was actually *not* secondary to administration of an analgesic, a finding that only highlights the problems with the quality of ExDS/ AgDS literature described in our study, and the associated challenges in accurately categorizing cases. Interestingly, if we had included this nonfatal case of restrained AgDS in our original analysis, it would have only strengthened the conclusion that ExDS is a diagnosis used when there has been restraint and a death, and when a person survives (even with restraint), they are more likely to be diagnosed with AgDS. It is noteworthy that, like other cited authors, Samuel et al. considered the term ExDS to be synonymous with AgDS, further supporting our assertion in our study that the two terms are often used interchangeably in the literature.

*de Boer et al.*:

*Schiavone et al. is cited as if it contributes one case, but the paper does not contain any case information.*

Response:

Schiavone et al. published two similarly titled ExDS papers in the same year, and the incorrect citation was included in the original article. The correct citation is *Involvement of the NADPH oxidase NOX2-derived brain oxidative stress in an unusual fatal case of cocaine-related neurotoxicity associated with Excited Delirium Syndrome* [15]. We thank de Boer et al. for their detailed review highlighting this minor citation error; however, the miscitation did not change the case count or categorization used for the analyses, or the results or conclusions of our study.

*de Boer et al.*:

*Stratton et al. is cited as if it contributes 18 cases, but the paper describes 214 cases of ExDS with restraint (196 non-fatal, 18 fatal)* [16]*. The non-fatal cases had the same restraint techniques used as the fatal cases. The 18 fatal cases were described in more detail, making them more suitable for case analysis, but by ignoring all survivors Strömmer et al. misrepresented the original data set.*

Response:

The 18 fatal ExDS cases from the Stratton et al. paper were included because they were adequately described for inclusion in the individual analysis. The non-fatal cases were **not** adequately described for individual data collection, and thus were not included in the analysis per the case selection criteria set forth in the “Methods” section of our paper. de Boer et al. ignored these criteria in their rush to call our methods a “misrepresentation” of the reviewed studies, which is, in and of itself, a misrepresentation of our study.

We thank de Boer et al. for spotting a minor transcription error in the “Mortality (%)” column of Table 4 of our study, which is the list of grouped studies that were not included for analysis. At some point during the multiple iterations and edits that our study underwent during peer-review, the error occurred undetected. A corrected earlier version of the table is included at the end of this response, as an *erratum* to the original study. As we noted, the group studies were of highly variable quality, with many lacking basic details about the subjects and circumstances of deaths that were noted, and that aside from listing the studies and some basic counts of study subjects and percentage of fatalities, no meaningful analysis could be performed on them. The error in Table 4 thus had no effect on the analyses or conclusions of our study.

Despite our gratitude for bringing the error in Table 4 to our attention and the opportunity to publish an *erratum* (as Table 1 in the Appendix), as well as pointing out the 3 minor citation errors, we posit that it is more customary to correspond with authors when such an error has been spotted. Instead, de Boer et al. claimed that our study was “fundamentally flawed,” while at the same time demonstrating an obvious lack of understanding of the fundamental design of the study.

As a final point, we found the title of the commentary from de Boer et al. “Scrutinizing the causal link between excited delirium syndrome and restraint…” to be misleading. The authors made no attempt to achieve what they promised in their title, instead choosing to hurl a litany of unsubstantiated and false allegations of flawed methods, improper conclusions, and general malfeasance in our direction. The detection of a few legitimate minor citation and formatting errors notwithstanding, de Boer et al.’s polemic consists of logical fallacies, misrepresentations, and a generally inaccurate portrayal of the purpose, methods, admitted limitations, and conclusions of our study. Even if the handful of individual cases for which de Boer et al. asserted a different categorization were to be accommodated and re-analyzed in the individual case analyses, the results would be essentially unchanged. There is no information presented by de Boer et al. that contradicts, in the slightest, our conclusion that in the absence of restraint, there is no evidence that ExDS is a stand-alone cause of death. de Boer et al. have managed to generate plenty of heat, but no light whatsoever on this topic.

## Appendix

**Table 1:** Erratum to Table 4 from Strömmer et al., depicting data from grouped studies on ExDS and AgDS.

First Author Name	Year	Number of cases		Data source	Restraint status known	Mortality (%)
		ExDS	AgDS			
Baldwin	2015	73	-	Police data	No	3
Cole	2018	-	49	Emergency dept	Yes	0
Ezaki	2016	2	-	Post-mortem	No	100
Grant	2009	21	-	Post-mortem	No	100
Gray	2007	-	31	Emergency dept	No	0
Hall	2015	86	-	Police data	No	1
Ho	2009	102	-	Post-mortem	Yes	100
Li	2019	31	-	Emergency dept	No	0
Mash	2009	90	-	Post-mortem	Yes	100
Michaud	2016	35	-	Post-mortem	No	100
Miner	2018	-	68	Emergency dept	No	0
Mo	2020	37		Emergency dept	Yes	0
Pollanen	1998	21	-	Post-mortem	Yes	100
Ross	1998	61	-	Post-mortem	Yes	100
Ruttenber	1997	58	-	Post-mortem	No	100
Southall	2008	19	5	Post-mortem	Yes	100
Strote	2014	43	-	Emergency dept	Yes	0
Strote	2006	28	-	Post-mortem	No	100
Vilke	2019	21	-	Emergency dept	No	0
